# A Case of Squamous Cell Carcinoma of the Lung That Produced Granulocyte Colony-Stimulating Factor and Interleukin-6

**DOI:** 10.1155/2013/325127

**Published:** 2013-04-24

**Authors:** Nobuhiro Takeuchi, Kento Yamamoto, Kazuyoshi Naba

**Affiliations:** ^1^Department of Internal Medicine, Kawasaki Hospital, Kobe, Japan; ^2^Department of Laboratory Medicine, Kawasaki Hospital, Kobe, Japan

## Abstract

A 77-year-old woman visited our institution complaining of general fatigue. Chest radiography revealed masses in the upper and middle lung fields. Pathological findings for an endoscopic biopsy specimen revealed squamous cell carcinoma. High-grade fever developed and blood analyses revealed sustained elevated white blood cell count and C-reactive protein levels. Cytokine production by tumor cells was suspected; both serum granulocyte colony-stimulating factor (117 pg/mL; normal: <57.5 pg/mL) and interleukin-6 (83.5 pg/mL; normal: <2.41 pg/mL) levels were high. Immunohistochemical examination of biopsy specimens showed positive staining with antigranulocyte colony-stimulating factor and anti-interleukin-6 monoclonal antibodies. Diagnosis of a tumor that produced granulocyte colony-stimulating factor and interleukin-6 was established. The patient was administered best supportive therapy since she was not eligible for surgical treatment because of her poor respiratory function. She died from interstitial pneumonia exacerbation two months after this diagnosis. We present a female with squamous cell carcinoma of the lung that produced granulocyte colony-stimulating factor and interleukin-6.

## 1. Introduction

Granulocyte colony-stimulating factor (G-CSF) is a cytokine associated with granulocytosis [1]. In recent years, it has been determined that leukocytosis in malignant tumors is caused by G-CSF production [1]. Malignant tumors that produce G-CSF are designated as G-CSF-producing tumors that are found in various organs including lung [2], liver [3], kidney [4], and gallbladder [5]. Interleukin-6 (IL-6) is a known promoter of G-CSF and causes fever and elevated C-reactive protein levels [6]. Malignant tumors that produced IL-6 are designated as IL-6-producing tumors. Here we present a case of lung squamous cell carcinoma that produced both G-CSF and IL-6.

## 2. Case Presentation

**Figure 1:**
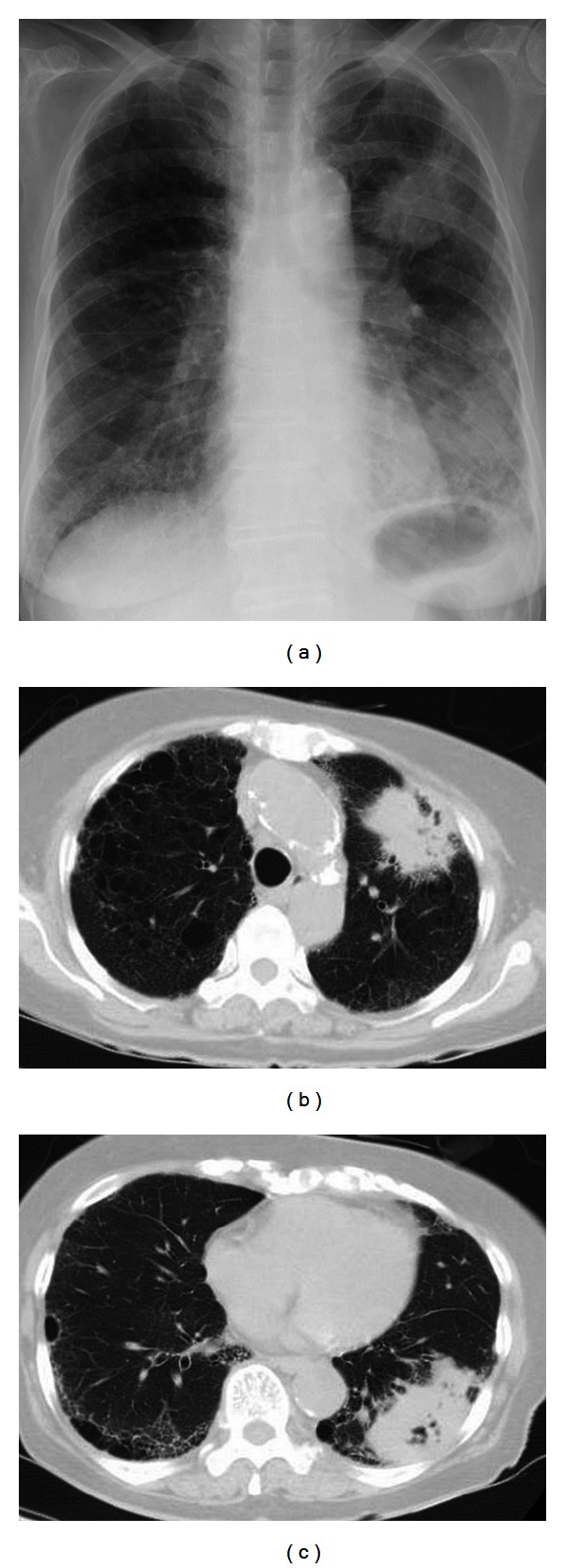
Chest radiography revealing a 5 cm mass in the upper lung field (a). Chest computed tomography (CT) revealing interstitial shadows in both lungs. Chest CT also revealed a 5.2 cm irregular mass with cavity and speculated margin in right S3 and a 7.6 cm irregular mass with cavity and air bronchogram sign, which are adjacent to the pleural walls ((b) and (c)).

A 77-year-old woman with a history of interstitial pneumonia and uveitis visited our institution complaining of general fatigue. Chest radiography revealed a mass in the upper lung fields (Figure 1(a)), and she was thus admitted for further examinations. 

Her blood pressure was 130/71 mmHg, heart rate was 106 beats/min, body temperature was 36.8°C, and O_2_ saturation was 96% in room air. She had a history of smoking 15 cigarettes per day for 55 years but no history of alcohol consumption. On initial clinical examination, her weight was of 50.8 kg, height was 158 cm, body mass index was 20.3 kg/m^2^, and BSA was 150 m^2^. Mild anemia was revealed in the palpebral conjunctiva. No abnormal murmur could be heard on auscultation although dry rales were heard in both lungs. 

Laboratory test results demonstrated a markedly elevated white blood cell count (266 × 10^2^/*μ*L; normal: 39–94 × 10^2^/*μ*L) with 81% neutrophils, mild anemia (red blood cell count: 322 × 10^4^/*μ*L; normal: 367–479 × 10^4^/*μ*L; hemoglobin: 9.8 mg/dL; normal: 11.5–14.9 mg/dL), elevated platelet count (56.9 × 10^4^/*μ*L; normal: 13–33 × 10^4^/*μ*L), mild renal dysfunction (serum creatinine: 0.96 mg/dL; normal: 0.47–0.79 mg/dL; serum urea nitrogen: 23.4 mg/dL; normal: 7–20 mg/dL), mild hyponatremia (133 mEq/mL; normal: 137–146 mEq/mL), mild hypercalcium (11.4 mg/mL; normal: 8.4–10 mg/dL), and markedly elevated C-reactive protein level (14.0 mg/dL; normal: 0–0.3 mg/dL). The tumor marker squamous cell carcinoma antigen was also elevated (5.5 ng/mL; normal: 0–1.5 ng/mL). 

**Figure 2:**
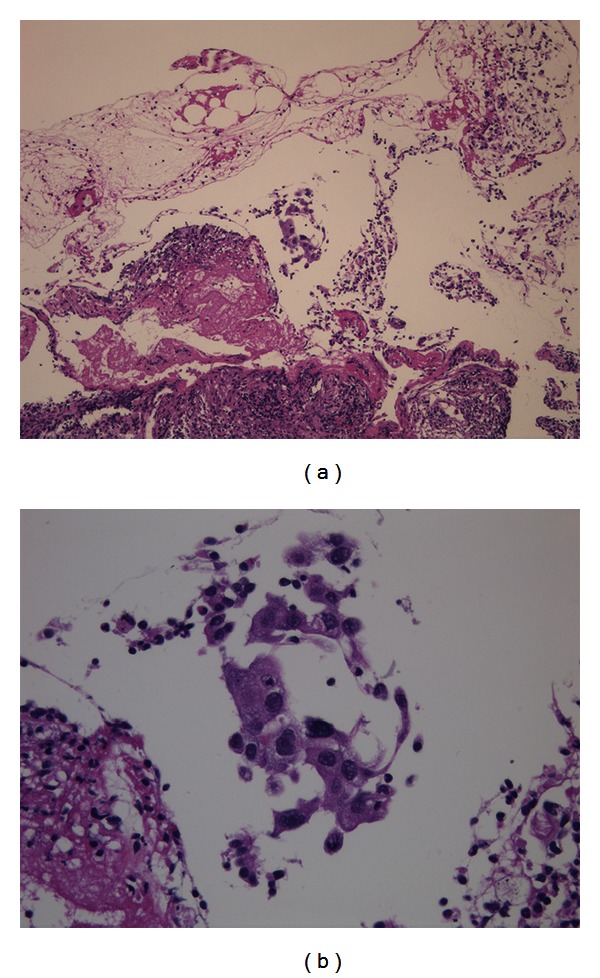
Pathological examination of biopsy specimens revealing squamous cell carcinoma (hematoxylin and eosin staining). (a) Low-power magnification and (b) high-power magnification.

Chest computed tomography (CT) revealed interstitial shadows in both lungs. Chest CT also showed a 5.2 cm irregular mass with cavity and speculated margin in the right S3 as well as a 7.6 cm irregular mass with cavity and air bronchogram sign, which were adjacent to the pleural walls (Figures 1(b) and 1(c)). A few nodules were found in the right lung. Left hilar and subcarinal lymph nodes were swollen. The pathological findings for an endoscopic biopsy specimen revealed squamous cell carcinoma (Figures 2(a) and 2(b)). Abdominal CT and brain magnetic imaging showed no lesion suggestive of metastasis. Radiographic findings confirmed T4N3M0 lung cancer of stage IIIB. 

After admission (Figure 4), high-grade fever developed and laboratory tests revealed sustained elevated white blood cell counts and C-reactive protein level. Therefore, the cooccurrence of a respiratory tract infection was suspected. The administration of antibiotics was initiated (sulbactam/ampicillin at 3.0 g/day for 6 days, and, subsequently, pazufloxacin at 1000 mg/day for 4 days). However, the elevated inflammatory marker levels and high-grade fever were not resolved. After administering nonsteroidal anti-inflammatory drugs (loxoprofen sodium hydrate at 180 mg/day), her temperature returned to normal. 

**Figure 3:**
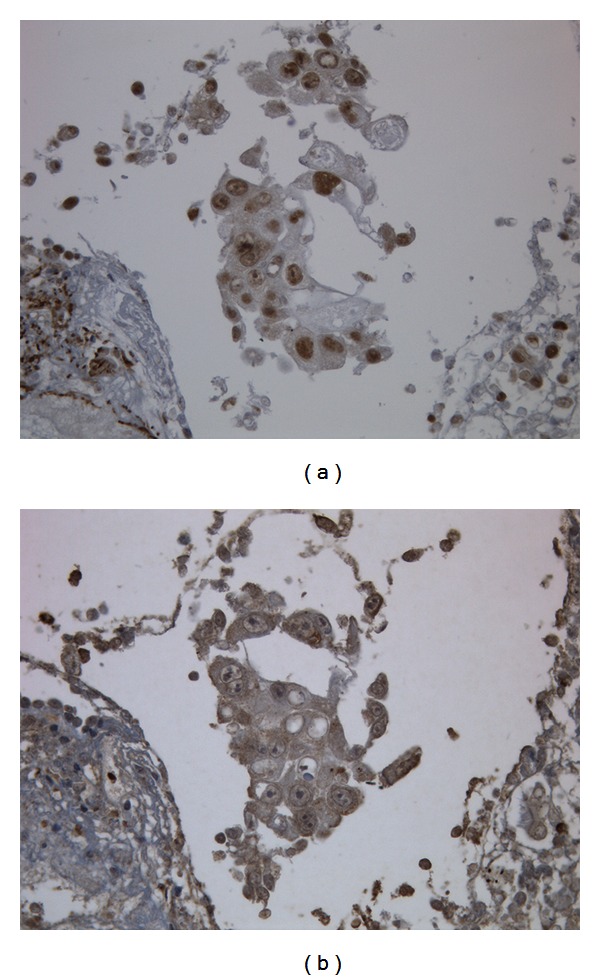
Immunohistochemical examination of tumor cells with an antigranulocyte stimulating factor antibody revealing nuclear membrane staining. (a) Immunohistochemical examination with an anti-interluekin-6 antibody revealing cytoplasmic and nuclear membrane staining (b).

**Figure 4:**
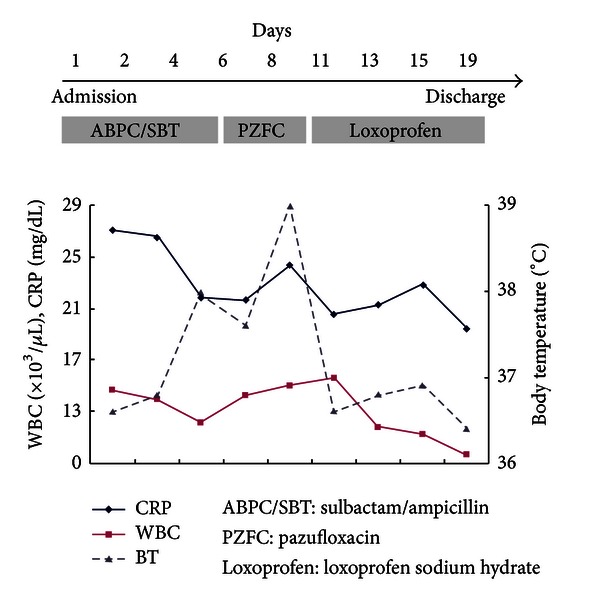
Clinical course after admission. Soon after admission, high-grade fever developed. Therefore, antibiotics administration was initiated (sulbactam/ampicillin at 3.0 g/day for 6 days, and, subsequently, pazufloxacin at 1000 mg/day for 4 days); the elevated inflammatory marker levels and high-grade fever were not resolved. After administering a non-steroidal anti-inflammatory drug (loxoprofen sodium hydrate at 180 mg/day), temperature returned to normal. White blood cell counts and C-reactive protein levels remained elevated.

The cause of fever and elevated inflammatory reaction levels was suspected as cytokine production by tumor cells. Therefore, serum G-CSF and IL-6 levels were measured, which showed that both serum G-CSF (117 pg/mL; normal: <57.5 pg/mL) and IL-6 (83.5 pg/mL; normal: <2.41 pg/mL) levels were high. Immunohistochemical examination of biopsy specimens showed positive staining with anti-G-CSF monoclonal and anti-IL-6 monoclonal antibodies (Figures 3(a) and 3(b)). Therefore, diagnosis of G-CSF- and IL-6-producing tumor was established. 

Although surgical treatment was considered, it was not indicated because her respiratory function was poor because of interstitial pneumonia. Chemotherapy was recommended, but she declined. Therefore, she was administered best supportive therapy. She was discharged 19 days after her admission, after which she visited our institution regularly. However, she was transferred by ambulance because of dyspnea, and she died of hypoxemia due to interstitial pneumonia exacerbation two months after diagnosis of lung cancer.

## 3. Discussion

Colony-stimulating factor (CSF) is one that promotes colony formation by leukocytes and other cells. G-CSF promotes granulocyte colony formation and is produced by macrophages, fibroblastic cells, and vascular endothelial cell. G-CSF also modulates differentiation and proliferation of granulocytes. In healthy individuals, G-CSF is present at very low levels in the blood circulation, and there is no correlation between circadian changes in serum G-CSF levels or leukocyte and granulocyte counts. 

Lung cancer is known to be accompanied paraneoplastic syndromes such as leucocytosis [7]. In 1966, Bardley and Metcalf [8] confirmed the concept of CSF as a humoral factor that promoted the proliferation of bone marrow stem cells. Robinson [9] provided evidence of CSF in the urine and serum of patients with malignant tumors. G-CSF is a cytokine associated with granulocytosis. In lung cancer with leukocytosis, tumor cells presumably produce G-CSF. 

In 1977, Asano et al. [1] reported that tumor cells could produce G-CSF and these tumors were designated as G-CSF-producing tumors. G-CSF-producing tumors are diagnosed when (1) there is marked leukocytosis without any other known cause, (2) there is elevated serum G-CSF level, (3) there is a decline in white blood cell counts after tumor resection, and (4) there is evidence of G-CSF production in tumor tissues.

In the past, evidence of G-CSF production in tumor tissues required methods showing *in vitro *colony formation, measuring G-CSF levels after transplanting tumor cells into nude mice [1], or detecting G-CSF mRNA in tumor cells [10]; therefore, their clinical application was quite complex. However, with improvements in enzyme immunoassay methods, it has become possible to measure serum G-CSF levels. Moreover, with improvements in anti-G-CSF monoclonal antibodies, it has become possible to provide evidence for G-CSF localization in tumor tissues by immunohistochemistry methods. 

Takahashi et al. [11] reported that, for the diagnosis of G-CSF-producing tumors, the confirmation of increased serum G-CSF levels and positive immunostaining with an anti-G-CSF antibody were sufficient. Among patients with G-CSF-producing tumors, some may present with fever or have elevated C-reactive protein levels because of other cause of leukocytosis [12]. G-CSF itself has no effect for causing fever or increasing C-reactive protein levels; therefore, the presence of inflammatory cytokines other than G-CSF is suggested. IL-6 is a promoter of G-CSF [6], and IL-6 produced by tumors can cause not only G-CSF production but also fever and elevated C-reactive protein levels. 

Currently, there is no clear definition for IL-6-producing tumors. Fujino et al. [13] provided evidence for production of IL-6 by measuring IL-6 levels produced by cultured tumor cells. In our case, in addition to the elevated serum IL-6 level, the confirmation of IL-6 in tumor cells from a biopsy specimen by immunostaining may have provided evidence for IL-6 production by tumor cells. Our case is significant because IL-6 production was demonstrated by pathological examinations.

Histologically, G-CSF-producing lung cancer comprises giant cell carcinoma, poorly differentiated adenocarcinoma, and squamous cell carcinoma. Moreover, G-CSF-producing lung cancer has poor prognosis with low-grade tumors and has the potential to invade into vessels and metastasize to lymph nodes. Even in resected cases, two-year mortality is as high as 15% [14]. 

Prognosis of G-CSF-producing tumors is poor, mortality over one year is as high as 12%, and the mean survival time is five months [15]. It is unclear if the reason for poor prognosis is the high malignancy potential of G-CSF-producing tumors or the G-CSF itself produced by tumors. 

Many G-CSF-producing tumors are suggested to be biologically highly malignant, poorly differentiated, and highly invasive to local lesions and vessels for the following reasons: (1) autocrine tumor growth because of the emergence of G-CSF receptors on tumor cells, (2) promotion of tumor growth and spread by proliferating neutrophils because of G-CSF, and (3) the inhibition of cellular immunity mediated by macrophages or natural killer cells by G-CSF. These mechanisms are presumably associated with tumor growth and spread [16].

Tumors that secrete autocrine growth factors show rapid progression. Moreover, the spread of tumor cells is closely associated with granulocyte macrophage colony-stimulating factor (GM-CSF), which is a known growth factor for macrophages and granulocytes. G-CSF-producing tumors probably produce cytokines other than G-CSF; thus, establishing a method to measure several cytokines simultaneously is desirable. 

G-CSF- and IL-6-producing lung cancer is comparatively rare because we could find only 5 reports concerning G-CSF- and IL-6-producing lung cancer in PubMed [17–21]. If the serum G-CSF and IL-6 level are measured commonly in lung cancer presenting with elevated white blood cell counts and C-reactive protein level, the more G-CSF and IL-6-producing lung cancer could be found. 

When leukocytosis without evidence of infection is found in solid cancer including lung cancer, the serum G-CSF-level should be measured to examine whether tumor cells secrete G-CSF. When G-CSF-producing tumor is diagnosed, the administration of antibiotics is unnecessary. If tumor can be resected, surgical treatment should be considered. 

Inoue et al. [20] reported a case of G-CSF- and IL-6-producing lung cancer showing elevated G-CSF and IL-6 level in tumor cells; although they showed the expression of G-CSF receptors on the surface of tumor cell, they could not show the expression of IL-6. In their case, although tumor cells secrete IL-6, IL-6 did not affect the proliferation of tumor cells. As in our case, we did not examine the expression of G-CSF receptors and IL-6 receptors; therefore, it is really unknown whether G-CSF and IL-6 are associated with the proliferation of tumor cells. 

## 4. Conclusion

We encountered a case of lung squamous cell carcinoma that produced both G-CSF and IL-6. When encountering patients with malignant tumors who present with elevated white blood counts and C-reactive proteins levels, serum G-CSF and IL-6 measurements and immunostaining tumor cells with anti-G-CSF and anti-IL-6 monoclonal antibodies should be considered for diagnosis of G-CSF- and IL-6-producing tumors.

## References

[1] Asano S, Urabe A, Okabe T, Sato N, Kondo Y (1977). Demonstration of granulopoietic factor(s) in the plasma of nude mice transplanted with a human lung cancer and in the tumor tissue. *Blood*.

[2] Kaira K, Ishizuka T, Tanaka H (2008). Lung cancer producing granulocyte colony-stimulating factor and rapid spreading to peritoneal cavity. *Journal of Thoracic Oncology*.

[3] Yamamoto S, Takashima S, Ogawa H (1999). Granulocyte-colony-stimulating-factor-producing hepatocellular carcinoma. *Journal of Gastroenterology*.

[4] Wang YC, Yang S, Tzen CY, Lin CC, Lin J (2006). Renal cell carcinoma producing granulocyte colony-stimulating factor. *Journal of the Formosan Medical Association*.

[5] Ikeda T, Ohgaki K, Miura M, Aishima S, Shimizu T, Maehara Y (2005). Granulocyte-colony stimulating factor-producing gallbladder cancer without recurrence more than 2 years after resection: report of a case. *Surgery Today*.

[6] Shannon MF, Coles LS, Fielke RK, Goodall GJ, Lagnado CA, Vadas MA (1992). Three essential promoter elements mediate tumour necrosis factor and interleukin-1 activation of the granulocyte-colony stimulating factor gene. *Growth Factors*.

[7] Heinemann S, Zabel P, Hauber HP (2008). Paraneoplastic syndromes in lung cancer. *Cancer Therapy*.

[8] Bradley TR, Metcalf D (1966). The growth of mouse bone marrow cells *in vitro*. *The Australian Journal of Experimental Biology and Medical Science*.

[9] Robinson WA (1974). Granulocytosis in neoplasia. *Annals of the New York Academy of Sciences*.

[10] Shimamura K, Fujimoto J, Hata JI (1990). Establishment of specific monoclonal antibodies against recombinant human granulocyte colony-stimulating factor (hG-CSF) an their application for immunoperoxidase staining of paraffin-embedded sections. *The Journal of Histochemistry and Cytochemistry*.

[11] Takahashi H, Andoh T, Kato T, Uehara T, Satoh N, Takeda B (1996). A case of plemorphic renal cell carcinoma producing G-CSF. *Journal of Japanese Society of Clinical Cytology*.

[12] Tsuyuoka R, Takahashi T, Sasaki Y (1994). Colony-stimulating factor-producing tumours: production of granulocyte colony-stimulating factor and interleukin-6 is secondary to interleukin-1 production. *European Journal of Cancer A*.

[13] Fujino M, Kimura H, Yamaguchi Y, Baba M, Iseki T, Sugita K (1992). A case of large cell carcinoma of the lung with marked leukocytosis and thrombocytosis producing colony stimulating factor (CSF). *Japanese Journal of Lung Cancer*.

[14] Nonami Y, Kondou J, Yamamoto A, Yamashiro T, Sasaguri S (2003). A case of G-CSF producing large cell carcinoma of the lung at first suspected to be pyothorax. *The Japanese Association for Chest Surgery*.

[15] Mizuguchi S, Inoue K, Iwata T (2005). The case of a lung adenocarcinoma producing granulocyte-colony stimulating factor (G-CSF) accompanied by arthritis. *The Japanese Association for Chest Surgery*.

[16] Tomizawa Y, Kuroiwa H, Suda T (1996). Rapid progression of adenocarcinoma of the lung in a patient with high levels of granulocyte colony-stimulating factor. *The Japanese Journal of Thoracic Diseases*.

[17] Matsuda E, Okabe K, Tao H (2011). Clinical assessment of granulocyte colony-stimulating factor producing lung cancer. *Kyobu Geka*.

[18] Matsuda E, Okabe K, Yagi T (2008). Granulocyte-colony stimulating factor producing tumor with high serum interleukin-6. *Kyobu Geka*.

[19] Sekido Y, Sato M, Usami N (2002). Establishment of a large cell lung cancer cell line (Y-ML-1B) producing granulocyte colony-stimulating factor. *Cancer Genetics and Cytogenetics*.

[20] Inoue M, Minami M, Fujii Y, Matsuda H, Shirakura R, Kido T (1997). Granulocyte colony-stimulating factor and interleukin-6-producing lung cancer cell line, LCAM. *Journal of Surgical Oncology*.

[21] Tsuyuoka R, Takahashi T, Sasaki Y (1994). Colony-stimulating factor-producing tumours: production of granulocyte colony-stimulating factor and interleukin-6 is secondary to interleukin-1 production. *European Journal of Cancer A*.

